# BMP4 Cooperates with Retinoic Acid to Induce the Expression of Differentiation Markers in Cultured Mouse Spermatogonia

**DOI:** 10.1155/2016/9536192

**Published:** 2016-10-04

**Authors:** Yongguang Yang, Yanmin Feng, Xue Feng, Shangying Liao, Xiuxia Wang, Haiyun Gan, Lixian Wang, Xiwen Lin, Chunsheng Han

**Affiliations:** ^1^State Key Laboratory of Stem Cell and Reproductive Biology, Institute of Zoology, Chinese Academy of Sciences, Beijing 100080, China; ^2^Graduate University of the Chinese Academy of Sciences, Chinese Academy of Sciences, Beijing 100101, China

## Abstract

Spermatogenesis is sustained by the proliferation and differentiation of spermatogonial stem cells (SSCs). However, the molecules controlling these processes remain largely unknown. Here, we developed a simplified high concentration serum-containing system for the culture of mouse SSCs. Analysis of SSCs markers and transplantation results revealed that the cultured spermatogonia retained stem cell characteristics after long-term* in vitro* propagation. Using this culture system, the expression and function of bone morphogenetic protein 4 (BMP4) were explored. Immunostaining showed that BMP4 was predominantly expressed in germ cells and that its level increased as spermatogenesis progresses. BMP4 receptors BMPR1A and BMPRII were present in spermatogonia, spermatocytes, and round spermatids. Moreover, despite the mRNAs of these two genes being present in mouse Sertoli cells, only BMPRII was detected by using Western blotting assays. While exogenous BMP4 by itself did not induce the expression of Stra8 and c-Kit, two marker genes of differentiating spermatogonia, a significant cooperative effect of BMP4 and retinoic acid (RA) was observed. Moreover, pretreatment of cultured spermatogonia with the BMP4 antagonist Noggin could inhibit RA-induced expression of these two marker genes. In conclusion, BMP4 may exert autocrine effects and act cooperatively with RA to induce the differentiation of spermatogonia* in vivo.*

## 1. Introduction

Mammalian spermatogenesis is a highly productive and organised process of cell proliferation and differentiation, resulting in the production of virtually unlimited number of spermatozoa throughout the life of the male [1]. This system roots in SSCs, which are capable of both self-renewal and differentiation dependent on the extrinsic and intrinsic cues [2]. Taking advantage of the discovery that GDNF stimulates SSC survival and proliferation* in vivo* [3], two groups first established the long-term cultures of mouse SSCs [4, 5]. In most studies, the initiation of SSCs cultures requires low concentration of foetal calf serum (FCS) in addition to several key growth factors [6]. Although a serum-free and feeder-free culture system has been established recently, the inclusion of serum products such as BSA and fetuin, which may contain other contaminated substances, not only resulted in variable passage timing and colony morphology but also raised the question whether a true chemically defined system was feasible for the culture of SSCs [7, 8]. Remarkably, several studies have demonstrated that SSCs can also be reprogrammed to ES-like pluripotent stem cells that contribute to the three embryonic germ layers with germ line transmission under certain culture conditions of high concentration of FCS without the introduction of exogenous genes [9–11]. However, the reprogramming efficiency and the reproducibility are low and the underlying mechanisms are unknown. In the present study, we report that SSCs could be cultured in embryonic stem cell (ESC) medium supplemented with GDNF and bFGF for as long as 33 passages (6 months) without the observation of ESC-like clones.

Bone morphogenetic proteins (BMPs), which belong to the TGF-*β* superfamily, are widely expressed during mouse embryogenesis and in adults and play key roles in male reproductive biology [12]. The TGF-*β* superfamily members function as homodimers or heterodimers by binding to heterogenic receptor complexes containing type I and type II serine-threonine kinase receptors [13], both of which are essential for signal transduction [13–18]. Bone morphogenetic protein 4 (BMP4) is known to be important for germ cell differentiation and survival [19–21]. In mouse, targeted knockout of the BMP4 gene results in failure of formation of primordial germ cells (PGCs) [22, 23]. BMP4 is also necessary for the localization of PGCs to genital ridge and the survival of the genital ridge [24]. In the postnatal testis, one report showed that BMP4 was expressed in Sertoli cells and stimulates the expression of c-Kit in cultured spermatogonia [25], whereas another study indicated that BMP4 was predominantly expressed in spermatogonia and RA initiates the resumption of spermatogenesis through the suppression of BMP4 expression in vitamin A-deficient (VAD) mice [19]. Hu et al. found that BMP4 mRNA is localized primarily in spermatocytes and in other cells, including Sertoli cells, to a less extent [26, 27]. These discrepancies warrant further clarification of the localization BMP4 in postnatal mouse testis as well as its function in spermatogenesis.

Germ cells in embryos are bipotential at the beginning, and their final fates are determined by RA produced by mesonephric duct [28, 29]. Retinoic-degrading enzyme CYP26B1 prevents germ cells from initiating meiosis in male mouse gonad during embryogenesis [28, 30]. In VAD mouse, only germ cells at early stages are present [19] and the differentiation of the aligned type A (A_al_) spermatogonia is inhibited [31–33]. Importantly, the capability of A_al_ spermatogonia to differentiate is restored upon administration of RA.

Given that both RA and BMP4 play important roles in various biological processes, it is not surprising that they interact* in vitro* and* in vivo* in several systems. For example, BMP2 and BMP4 have been shown to interact with RA signalling to induce apoptosis of P19 embryonic carcinoma cells [34, 35]. In foetal vertebrate limbs, BMP signalling is also known to mediate RA-induced interdigital cell apoptosis [36]. While it is evident that both RA and BMP4 are necessary for the male reproductive capacity, whether these signalling pathways interact with each other in the germ cells is yet to be determined. In this study, we examined the expression of BMP4 and its receptors in postnatal germ cells and Sertoli cells. Furthermore, we aimed to investigate whether BMP4 and RA could cooperatively regulate the expression of Stra8, a gene that is stimulated by RA for the initiation of meiosis [37] and c-Kit whose expression is activated in differentiating spermatogonia [38] by using cultured mouse spermatogonia.

## 2. Materials and Methods

### 2.1. Reagents and Experimental Animals

Recombinant rat GDNF and bFGF, recombinant human BMP4 factors, and mouse anti-BMP4 primary antibody were from R&D Systems (Minneapolis, MN, USA). RA was purchased from Sigma (Sigma-Aldrich). Rabbit anti-human RET (SC-167) and GFRa-1 (H-70), goat anti-human BMPRII (G-17) and *β*-actin polyclonal antibodies, FITC- and TRITC-conjugated anti-rabbit, and goat secondary antibodies were from by Santa Cruz (Santa Cruz, CA, USA). Mouse against PLZF mAb, DNase I, and collagenase type IV were from Calbiochem (Merck KGaA, Darmstadt, Germany). Goat against human vimentin polyclonal antibody and FCS (ES cell-qualified) and ESGRO LIF were from Chemicon (Billerica, MA, USA). Rabbit against BMPR1A and DAZL antibodies were from Abcam, and rabbit against p-Smad1/5/8 antibody was from Cell Signalling Technology (Beverly, MA, USA). High glucose DMEM was from Hyclone, while DPBS and 0.25% Trypsin-EDTA were from Gibco (Carlsbad, CA, USA). DBA/C57 F1 mice were obtained from Beijing Weitong River Laboratory Animal Inc., China. Ub-eGFP-lentivirus was bought from Shanghai GeneChem Company (GeneChem, Shanghai, CN). All protocols were approved by the Animal Care and Use Committee of the Institute of Zoology, Chinese Academy of Sciences.

### 2.2. Cell Culture Conditions

ESC medium consisted of DMEM (4.5 g/liter glucose) supplemented with 15% FCS, 1x NEAA, 50 *μ*M 2-mercaptoethanol, 1x sodium pyruvate, 2 mM L-glutamine, 100 units/mL penicillin, 100 units/mL streptomycin, and 10^3^ units/mL LIF, stored at 4°C and could be used for up to 3 weeks. 40 ng/mL GDNF and 10 ng/mL bFGF were added to the ESC medium shortly before use to make the SSC medium. Mouse embryonic fibroblast (MEF) cells were isolated from 13.5-day CD1 mouse embryo and cultured in DMEM medium supplemented with 10% FCS, 50 *μ*M 2-mercaptoethanol, 2 mM L-glutamine, and antibiotics. For preparation of feeder layer, MEF cells were treated with mitomycin C (10 *μ*g/mL, Roche) for 2.5 hours and then plated at a density of 5 × 10^4^ cells/mL in wells precoated with 0.2% gelatin (Sigma-Aldrich). All cells were maintained at 37°C with an atmosphere of 5% CO_2_. MEF cells can be used after 24 hours of plating and are viable for one week.

### 2.3. Isolation and Collection of Mouse Spermatogonia

Spermatogonia from 4-5-day-old pup mice were prepared by following the two-step enzymatic digestion protocol [39] with some modifications. Briefly, decapsulated testis tissue was treated with 1 mg/mL collagenase type IV and 1 mg/mL DNase I for 5 min with gentle agitation in DPBS and then centrifuged at 50 ×g for 2 min followed by 3 washes with DPBS to remove the interstitial cells. Collected specimens were then treated with 0.25% Trypsin-EDTA and 1 mg/mL DNase I for another 5 min in 37°C with gentle agitation. Subsequently, FCS was added at a final concentration of 15% to terminate the digestion. Cells were then washed twice by centrifugation at 600 ×g for 5 min in DPBS. The pellet was resuspended in SSCs growth medium, plated on 0.2% (w/v) gelatin-coated tissue culture dish with a density of 2 × 10^5^ cells per cm^2^ and cultured for 36–48 hours. Then, the medium was removed and cells were washed once with DPBS, pipetted gently to detach the spermatogonial germ cells from the attached somatic cells, and centrifuged at 600 ×g for 5 min. Germ cells were resuspended in SSCs culture medium and plated on the MEF feeder layer.

### 2.4. BMP4 and RA Treatment of Cultured Spermatogonia

About 2000 cells were cultured on laminin-coated plate with 15% or 1% FCS-containing medium in the presence of GDNF and bFGF for 24 hours and serum starvation for 6 hours before BMP4 treatment. Spermatogonia were stimulated with 100 ng/mL BMP4 for 1 hour and fixed with 4% PFA for further analysis. About 2 × 10^5^ cells were cultured on MEF and treated the same way and protein was collected for Western blotting analysis. For the detection of BMP4- and/or RA-induced spermatogonial differentiation, about 2 × 10^4^ spermatogonia were plated on MEF feeder and treated with BMP4 for 24 hours and then RA was added in the presence of BMP4 to continue culture for indicated time. Cells were collected for RNA preparation and real-time PCR analysis.

### 2.5. Transplantation and Analysis

DBA/ICR F1 hybrid male mice were used as recipients. First, 4–6-week-old mice were treated with 40 mg/kg busulfan to deplete the endogenous germ cells in the testis [40, 41]. For lentivirus infection, lentivirus was added into the medium at 20 MOI (multiplicity of infection) with 5 *μ*g/mL polybrene. The medium was changed at 8–10 hours after transfection and the cells were maintained* in vitro* for proliferation. For testis transplantation, approximately 40 *μ*L of virus transfected donor cells suspension (6 × 10^5^ cells) was injected into the testis seminiferous tubules of the recipient mouse through the efferent duct [39], filling about 70% of the tubules. Six months after transplantation, testes were collected for analysis.

### 2.6. Preparation of Spermatocyte, Round Spermatid Cell, and Sertoli Cells

Highly enriched mouse spermatocyte and round spermatid cells were isolated from adult mouse testicular cell suspensions using cell-size fractionation by sedimentation through a bovine serum albumin gradient at unit gravity following Belle's protocol (see Figure S4A–C in Supplementary Material available online at http://dx.doi.org/10.1155/2016/9536192) [42]. The Sertoli cells were cultured by following the procedure published previously [43].

### 2.7. Immunostaining

For IFA staining, cells were fixed with 4% PFA followed by treatment with 0.1% Triton X-100 and blocked with BSA. Samples were then incubated at 4°C with indicated primary antibodies overnight. Nonspecific IgG was used as negative control. Then, samples were washed thrice with PBST and incubated with FITC- or TRITC-conjugated secondary antibody. Nuclei of the cells were stained with DAPI. For immunostaining with paraffin sections, after deparaffinization and antigen retrieval, following the protocol as described above. Fluorescent images were captured under Olympus IX 71 microscope, and images were combined and processed in Adobe Photoshop 7.0.

### 2.8. RNA Extraction, RT-PCR, and Real-Time RT-PCR

Total RNA was extracted using TIANGEN RNA Pre Micro kit according to the manufacturer's instruction. Reverse transcription was performed using oligo (dT) priming and M-MLV reverse transcriptase (Promega) following the manufacturer's instructions. PCR primers were listed in Table 1. PCR products were separated by electrophoresis on 1.5% agarose gels. Real-time RT-PCR were performed on ABI 7500 sequence detection system and analysed with ABI 7500 software.

### 2.9. Western Blotting Analysis

Cells were harvested and homogenised at 4°C in lysis buffer containing preformed protease inhibitors mixture (P8340, Sigma). Total proteins were transferred to polyvinylidene difluoride membranes after SDS-PAGE, blocked with 5% fat-free milk then hybridized with primary antibodies. After hybridization with HRP-conjugated secondary antibody, immune-complexes were detected using Supersignal West Pico detection reagent (Pierce).

### 2.10. Statistical Analyses

All experiments were repeated at least thrice unless otherwise stated. Densitometry of Western blotting was conducted using the Quantity One software with GAPDH as internal control. Values were presented as mean ± standard deviation (SD) of three separate experiments. Statistically significant differences (*P* < 0.05 or *P* < 0.01) among groups were determined by ANOVA and Tukey posttests using SPSS statistical software (SPSS Inc., Chicago, IL, USA).

## 3. Results

### 3.1. Establishment of a Simplified Long-Term Culture System for Mouse Spermatogonia

**Figure 1:**
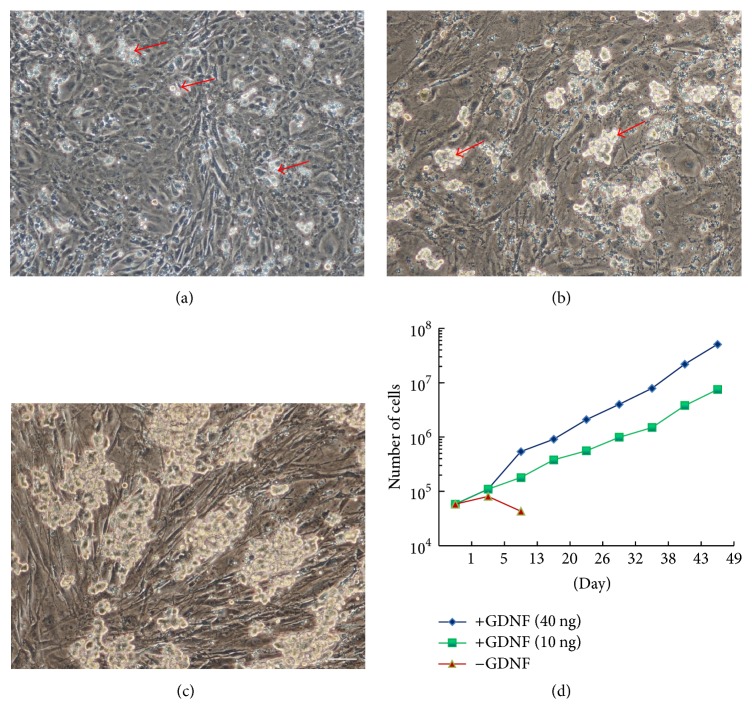
Establishment of mouse spermatogonial cultures* in vitro*. (a) Single spermatogonium or small spermatogonial clumps (red arrow) were observed 36 hours after culture* in vitro*. (b) At Passage 3 (P3), cells began to form stable colonies (red arrow) on MEF. (c) Spermatogonial clumps formed on MEF at day 180* in vitro*. (d) Proliferation curves of spermatogonial cultures. Within 7-week period, spermatogonia expanded approximately 750-fold with 40 ng/mL GDNF and 500-fold with 10 ng/mL GDNF. Scale bar: 20 *μ*m.

To establish SSC cultures* in vitro*, testicular cells were isolated using a modified two-step enzymatic digestion method [39]. After 24–36 hours of incubation in ESC medium supplemented with 40 ng/mL GDNF, somatic cells adhered tightly to the gelatin-coated dish and the germ cells, distinguished by their large size, attached loosely to the somatic cells. Germ cells at this stage were considered as Passage 0 (P0) of the spermatogonia culture (Figure 1(a)). The germ cells were then transferred to mouse embryonic fibroblast (MEF) feeder cells in a second culture dish by gentle pipetting; germ cells at this stage were cultured in ESC medium supplemented with GDNF, FGF2, and LIF and were considered as Passage 1 (P1). Cells at later passages were maintained in the same medium. Although most somatic cells can be removed at this step, some of them still remained. Therefore, spermatogonial clumps were mechanically collected and plated onto fresh feeder layers for the first 2-3 passages (Figure S1A). At this stage, the remaining somatic cells adhered tightly to the feeder layer and germ cells formed small clumps (Figure 1(b)). From the third passage, germ cells were passaged using 0.25% Trypsin-EDTA in a 1 : 3 dilution once every 5–7 days onto MEF feeders. By 2-3 weeks, cultures reached a relatively steady state and continued to generate clumps of similar morphology. Two strains of spermatogonia have been passaged for 6 months (P33)* in vitro* (Figure 1(c)). This method could be used to establish cultures from 4-5-day-old animals of the DBA/C57 F1 or DBA/ICR F1 mice. Consistent with previous reports, GDNF was indispensable for the long-term culture of mouse spermatogonia and it, at a concentration of 10 ng/mL in the medium, was enough to support the long-term proliferation of spermatogonia (Figure 1(d)).

### 3.2. Characterization of Cultured Mouse Spermatogonia

**Figure 2:**
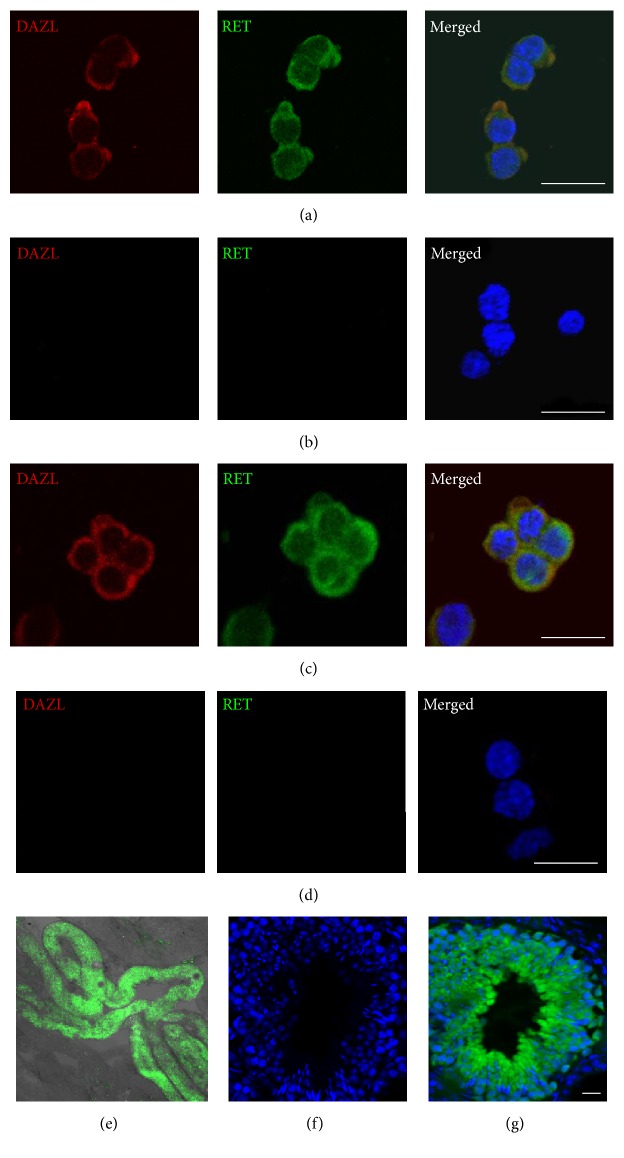
Characterization of cultured spermatogonia by examining SSC marker gene expression and transplantation assays. (a-b) P3 (6 days* in vitro*) culture of spermatogonia stained with antibodies against DAZL and RET (a) and nonspecific IgG (b). (c-d) P33 (180 days* in vitro*) culture of spermatogonia stained with antibodies against DAZL and RET (c) and nonspecific IgG (d). (e) Fluorescent image of seminiferous tubules of a recipient testis at 6 months after receiving Ub-EGFP-lentivirus infected spermatogonia. Note the extensive recolonization of green cells in the seminiferous tubules. (f-g) Cross-section of a seminiferous tubule recolonized by green germ cells at various differentiating stages. Scale bar: 20 *μ*m.

Immunostaining was employed to characterize the cultured spermatogonia. Clumps formed in early (P3) (Figures 2(a) and 2(b)) and long-term cultures (6 months* in vitro*, P33) (Figures 2(c), 2(d), S1B, and C) were positive for DAZL, a germ cell marker [44], and RET, a SSCs marker [45]. To further confirm the stem cell activity of these cultured spermatogonia, 6 × 10^5^ GFP-expressing SSCs were injected into the testes of busulfan-treated adult recipient mice. Six months after transplantation, the recipient mice were sacrificed for analysis. The green spermatogonia could colonize the seminiferous tubules and restore spermatogenesis (Figure 2(e)). Importantly, the green spermatogonia in the seminiferous tubules could differentiate into germ cells at different stages (Figures 2(f), 2(g), and S2). These results confirmed the existence of SSCs in the spermatogonial cultures using the mouse ESC culture medium supplied with GDNF and FGF2.

### 3.3. Expression of BMP4 and Its Receptor Subunits in Germ Cells

**Figure 3:**
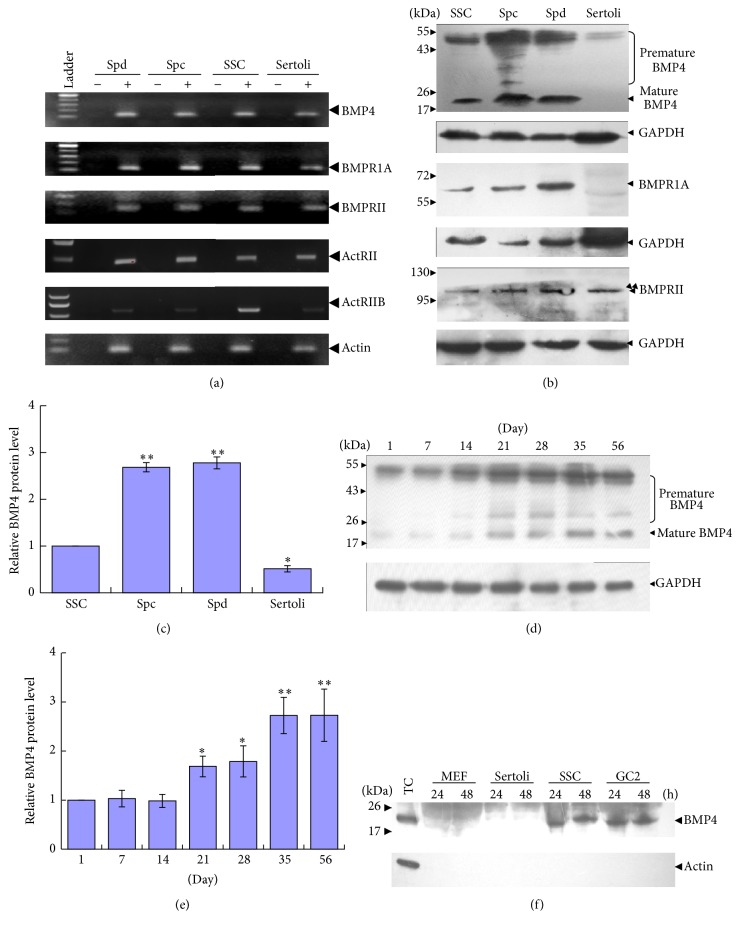
Expression of BMP4 and its receptor subunits in testicular cells. (a) RT-PCR analyses of the expression of* BMP4* and its receptors* BMPR1A*,* BMPRII*,* ActRII*,* ActRIIB,* and actin in isolated spermatocytes (Spc) and round spermatids (Spd), as well as in cultured spermatogonia (Spg) and Sertoli cells (Sertoli). RNA samples without RT but with PCR amplification were used as negative control. (b-c) Western blotting analysis of the expression of BMP4 and its receptors in 4-5-day-old mouse Spg, adult Spc and Spd, and Sertoli cells (b) and quantification of BMP4 protein in these cells (c). The premature and mature forms of BMP4 were labelled. (d-e) Western blotting analysis of BMP4 protein in mouse testis at indicated development stages (d) and quantification (e). (f) Western blotting analyses of secreted BMP4 in the medium from different cell cultures. Adult mouse testicular cells lysate (TC) was used as positive control. The data is presented as means ± SD from at least three independent experiments. Statistically significant differences among groups are indicated by asterisks, ^*∗*^ *P* < 0.05 or ^*∗∗*^ *P* < 0.01.

Using the simplified spermatogonial culture system, we decided to study the function of BMP4 in SSC differentiation* in vitro*. RT-PCR analysis first showed the mRNAs of* BMP4* and its receptors* BMPR1A*,* BMPRII*,* ActRII,* and* ActRIIB* were present in cultured spermatogonia, Sertoli cells, and isolated adult spermatocytes as well as round spermatids (Figure 3(a)). Western blotting results further revealed that the premature and mature forms of BMP4 protein were predominantly expressed in spermatogenic cells, especially in adult spermatocytes and round spermatids (Figures 3(b) and 3(c)). Interestingly, BMPRII protein but not BMPR1A was present in Sertoli cells, which implies the absence of an intact BMP4 signalling pathway in these cells. Moreover, Western blotting analysis also showed the increased BMP4 protein expression as mouse testis development progresses through 1 to 56 days after birth (Figures 3(d) and 3(e)).

To test whether cultured spermatogonia could secrete BMP4 factor into culture medium, the media of MEF, Sertoli cell, spermatogonia, and an immortalised preleptotene spermatocyte cell line GC-2 [46] were collected for Western blotting analysis (Figure 3(f)). The results showed that cultured spermatogonia and GC-2 but not MEF and Sertoli cells could secrete BMP4 protein into the medium.

**Figure 4:**
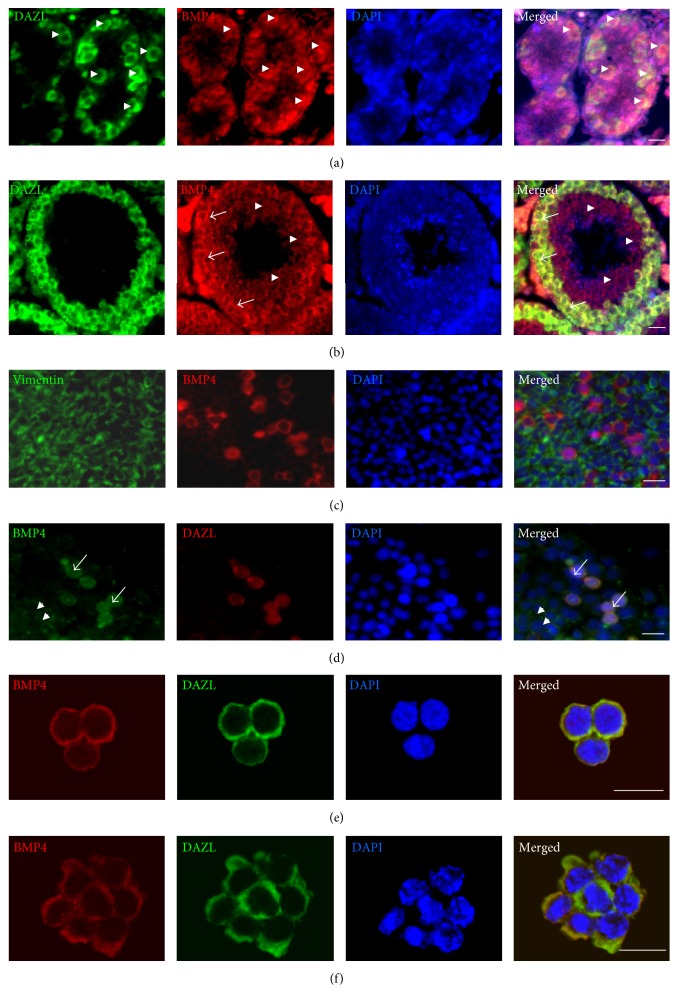
BMP4 protein is mainly expressed in mouse germ cells. (a) IFA analysed the localization of BMP4 and DAZL in 7-day mouse testis section (arrow head). (b) In adult mouse testis section, BMP4 was predominantly expressed in spermatocytes (arrow) and round spermatids (arrow head). (c-d) In testicular cells isolated from 4-5-day mice, BMP4 was predominantly expressed in germ cells (arrow) and, to a less extent, in Sertoli cells (arrow head). (e-f) BMP4 was also expressed in P3 (e) and P33 (f) cultured spermatogonia. Scale bar: 20 *μ*m.

The expression of BMP4 in mouse testis sections, cultured testicular cells, and spermatogonia was subsequently examined by immunofluorescence staining. In 7-day-old mouse testis sections, BMP4 protein was mainly localized in DAZL-positive germ cells (Figure 4(a), arrow head), while, in adult mouse testis sections, BMP4 was predominantly expressed in spermatocytes (Figure 4(b), arrow) and round spermatids (Figure 4(b), arrow head). Surprisingly, in isolated 4-5-day mouse testicular cells, BMP4 was mainly coexpressed in DAZL-positive cells (Figures 4(c) and 4(d), arrow) and to a less extent in Sertoli cells (Figure 4(d), arrow head). BMP4 protein was also expressed in P3 (Figure 4(e)) and P33 (Figure 4(f)) cultured spermatogonia. These results indicate that BMP4 is mainly produced by germ cells in mouse testis.

### 3.4. Cultured Spermatogonia Respond to BMP4 Stimulation with Smad1/5/8 Activation

**Figure 5:**
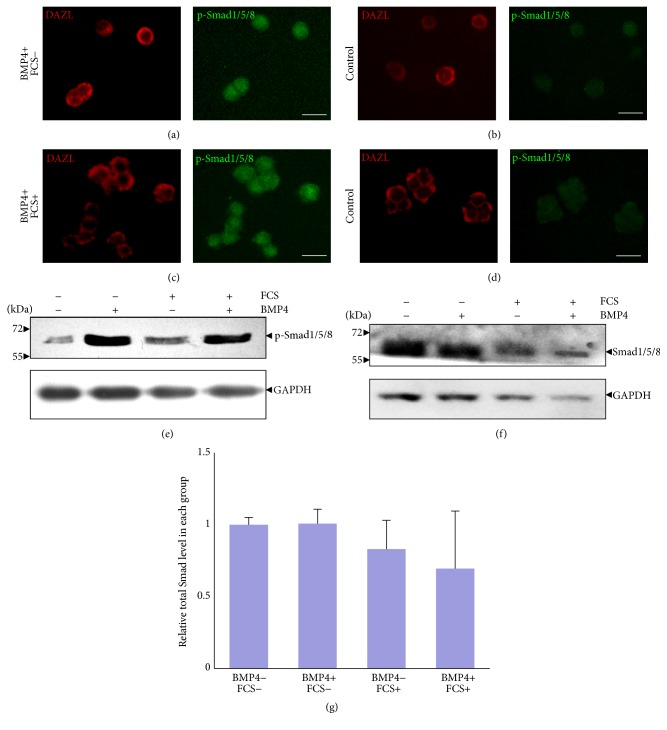
BMP4 activates Smad signalling pathway in cultured spermatogonia. (a-b) IFA analyses of spermatogonia cultured in serum-free medium using antibodies against phosphorylated Smad1, Smad5, and Smad8 in the presence of BMP4 (a) or control (b). (c-d) IFA analysis of spermatogonia cultured in 15% serum-containing medium in the presence of BMP4 (c) or control (d). (e-f) Western blotting analysis of phosphorylated (e) and total (f) Smad1/5/8 in spermatogonia under different culture conditions. (g) Relative total Smad1/5/8 level in each group in (f) after normalization to the internal control. Scale bar: 20 *μ*m.

Previous studies reported that BMPs induce the phosphorylation of R-Smads (Smad1, Smad5, and Smad8) to exert its function [47]. To evaluate the response of cultured spermatogonia to BMP4 stimulation in the current culture system, cells were treated with 100 ng/mL BMP4 factor for 1 hour. Immunofluorescent assay (IFA) revealed that BMP4 could activate and induce nuclear accumulation of phosphorylated Smad1/5/8 in both serum-free (Figures 5(a) and 5(b)) and 15% serum-containing (Figures 5(c) and 5(d)) medium. Western blotting analysis further confirmed the IFA results while the p-Smad1/5/8 level was significantly increased after BMP4 treatment; the total proteins level was not clearly changed when normalized to the internal GAPDH control (Figures 5(e)–5(g)). Together, these results showed the existence of an intact BMP4 signalling pathway in cultured spermatogonia.

### 3.5. BMP4 Cooperates with RA in the Induction of Stra8 and c-Kit Expression in Cultured Spermatogonia

**Figure 6:**
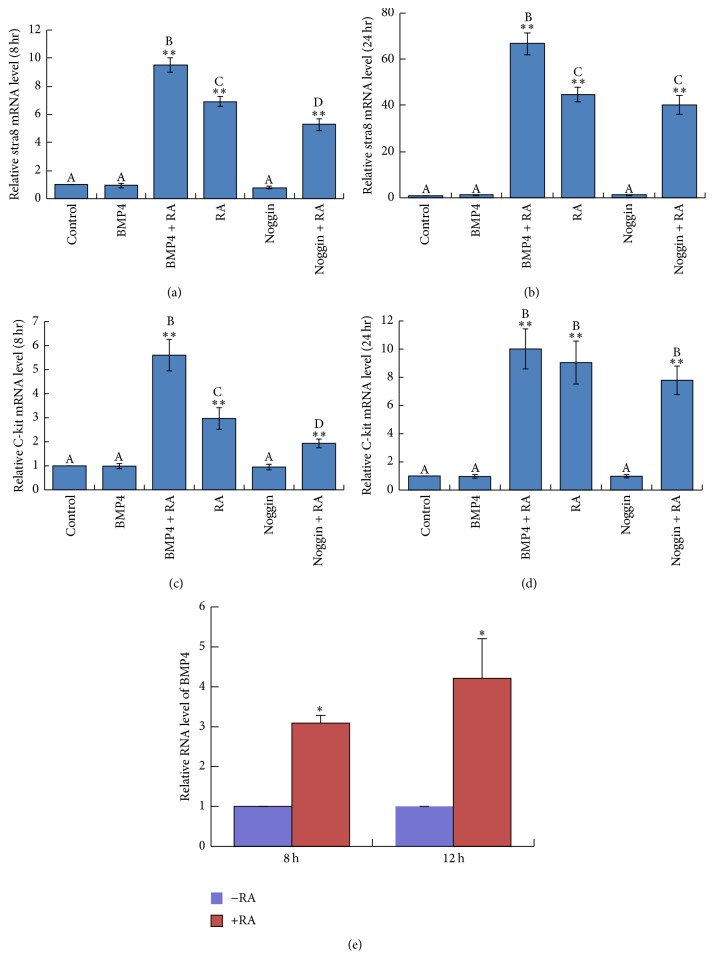
BMP4 induces the expression of Stra8 and c-Kit cooperatively with RA in cultured spermatogonia. (a-b) Real-time PCR analysis of Stra8 expression in cultured spermatogonia pretreated with BMP4 or Noggin and followed by RA stimulation for 8 hours (a) or 24 hours (b). (c-d) Real-time PCR analysis of c-Kit expression in cultured spermatogonia pretreated with BMP4 or Noggin and followed by RA stimulation for 8 hours (c) or 24 hours (d). (e) Real-time PCR analysis of BMP4 mRNA expression in cultured spermatogonia followed by RA alone stimulation for 8 hours and 12 hours. The columns were labelled with A, B, C, and D, which means values are significantly different (*P* < 0.05) among differently labelled groups; ^*∗*^ *P* < 0.05 or ^*∗∗*^ *P* < 0.01.

We next explored the differentiation-inducing function of BMP4 in cultured spermatogonia. Real-time PCR analyses were first employed to evaluate the expression of Stra8 and c-Kit in these cells treated with BMP4. Unexpectedly, BMP4 treatment for either 24 or 48 hours did not increase the expression of Stra8 and c-Kit in cultured spermatogonia (Figure S3A–D). Since RA is necessary for spermatogonial maturation and entry into meiotic prophase in postnatal testes [48, 49], we then analysed the effect of RA treatment on the expression of these differentiation markers. As anticipated, RA treatment could significantly increase Stra8 and c-Kit expression compared with that in control group (Figures 6(a)–6(d)). Intriguingly, pretreatment of spermatogonia with BMP4 for 24 hours and then stimulation with RA, the expression of Stra8 was significantly increased at 8 and 24 hours compared with the RA only groups (Figures 6(a)-6(b)). Similarly, the expression of c-Kit in the BMP4 plus RA group was also significantly higher than that in the RA only group at 8 hours after RA stimulation (Figure 6(c)). Importantly, pretreatment of spermatogonia with Noggin, a BMP4 antagonist [50, 51], could clearly reduce RA-induced Stra8 (Figure 6(a)) and c-Kit (Figure 6(c)) expression compared with RA only groups at 8 hours after stimulation. Finally, real-time PCR method was employed to evaluate whether RA treatment could alter the expression of BMP4 in cultured spermatogonia (Figure 6(e)). The results showed that BMP4 expression was significantly promoted by RA stimulation. This finding is consistent with the Western blots results in Figure 3(d), which showed that BMP4 is higher in more differentiated germ cells. These results suggest that BMP4 could act cooperatively with RA in inducing the expression of spermatogonial differentiation markers Stra8 and c-Kit.

## 4. Discussions

Researchers have long dreamed of producing sperm in culture dishes not only to correct certain types of male infertility but also to study the mechanism of spermatogenesis in a more convenient way [52]. There are two major obstacles for the accomplishment of this goal—the proliferation and induced differentiation of SSCs* in vitro*. The medium for spermatogonial culture described in current standard protocols contained many components, the roles of which have not been fully elucidated [4, 5]. Some studies claimed that the quality of some agents, such as serum, BSA, and lipid, are critical for the success of SSC cultures [5–7, 53]. Unfortunately, there is no guarantee to get the right batches of these agents commercially; therefore quality control of the culture system is troublesome. The serum-free and feeder-free culture system reported recently still uses serum-derived products such as BSA and fetuin, the possible contaminants in which remain as concerns for mechanistic studies and potential applications [8]. Since products from serum are inevitable, we decided to establish a simplified system, which included high concentration serum but not the other components such as BSA and lipids. We used DMEM supplemented with 15% FCS, three growth factors (LIF, GDNF, and bFGF), and some common nongrowth factor components, such as nonessential amino acids, glutamine. This is equivalent to the mouse ESC culture medium supplemented with GDNF and bFGF. Long-term spermatogonial cultures could be routinely established, with a maximum culture period of 33 passages over 6 months. The long-term cultured spermatogonia contained stem cells as indicated by transplantation assays. Most studies use low concentration serum (0.04–1%) for spermatogonial culture [4, 6, 53, 54] and some even report a detrimental effect of serum on SSCs [5, 55] while others claimed the appearance of ESC-like clones in ESC medium containing high concentration serum. Since we were able to culture SSCs with high concentration serum, we conclude that it may be the quality instead of the concentration of serum that determines the success of SSCs culture. Moreover, we never observed any ESC-like colonies during our whole experimental period, suggesting that this reprogramming process is indeed a rare event. In summary, our system is a much simplified one compared with other systems while it is comparable with the others in terms of GDNF dependency, long-term culture period, and the maintenance of stem cell activities.

While several secreted protein factors have been reported to enhance the proliferation of SSCs [4, 5, 56–59], the spermatogonia differentiation-inducing factors have been poorly defined. The expression of BMP4 in testes is still poorly defined. While one study showed that BMP4 mRNAs were detected in Sertoli cells of the 4–7 dpp mice but not in spermatogonia at the same stage, another one showed that it, in adult mouse testis, was predominantly expressed in early stage germ cells, including spermatogonia and early spermatocytes, but absent from Sertoli cells [19, 25]. Hu et al. found that BMP4 mRNA was localized in pachytene spermatocytes and, to a less extent, in other germ cells [26]. In the present study, we confirmed that BMP4 mRNAs are present in germ cells including spermatogonia, spermatocytes, and round spermatids as well as in the somatic Sertoli cells, while the protein was predominantly expressed in adult germ cells and marginally in Sertoli cells. Therefore, the expression of Bmp4 in testicular cells is developmentally regulated and the major cell source changes from Sertoli cells to germ cells, and its low level expression in certain cell types may be neglected by previous studies due to the use of insensitive detecting methods.

The TGF-*β* superfamily members function as homodimers or heterodimers by binding to heteromeric receptor complexes that contain type I and type II serine-threonine kinase receptors [26]. The study by Pellegrini et al. showed that mRNAs of BMP4 receptors* BMPR1A* and* BMPRII* were only detectable in 4- and 7-day-old mouse spermatogonia [25]. In the present study, we detected the expression of BMPR1A and BMPRII at both mRNA and protein levels in neonatal mouse SSCs, adult spermatocytes, and round spermatids. While* BMPR1A*,* BMPRII*,* ActRII,* and* ActRIIB* mRNAs expression were also detected in 4-5-day-old mouse Sertoli cells, BMPRII but not* BMPR1A* protein was detected by Western blotting. Pellegrini et al. showed that mRNAs of BMPRIA could not be detected by Northern blotting in the Sertoli cells of 4 dpp mice [25]. It is likely that BMPRIA mRNA level is very low and can only be detected by RT-PCR but not Northern blotting and consequently its protein cannot be detected by Western blotting readily. Taken together, it appears that, in adult mice, BMP4, which is majorly produced by germ cells, executes an autocrine function by binding to its receptor complex on germ cells.

BMP4 is well-documented to be necessary for the generation and survival of PGCs and might therefore be involved in the regulation of spermatogenesis [22, 24, 25, 60]. Whereas BMP4 alone is unable to induce the formation of PGCs, BMP8b is required for BMP4-induced PGC formation in cultured epiblasts [61]. In human endometrial stromal cells, TGF-*β* cooperates with RA to induce the expression of VEGF [62]. For VAD mouse, retinol, probably by being converted to its active metabolite RA, initiates the resumption of spermatogenesis through the suppression of BMP4 expression in spermatogonia [19]. In the immune system, synergy between RA and TGF-*β* induced Foxp3^+^ T cells at least 3 times higher than those induced by TGF-*β* alone [63]. A previous study also showed that RA and BMP4 synergistically induced the apoptosis of P19 embryonic carcinoma cells [64, 65]. These studies indicate that the cooperative interactions of RA and TGF-*β* family members are involved in diverse developmental processes.

An important observation in the present study was that BMP4 and RA cooperatively induced the mRNA expression of Stra8 and c-Kit, two genes involved in spermatogonial differentiation and meiosis initiation [66, 67]. One previous study reported that BMP4 treatment of germ cell-enriched cultures from day 4 mouse testes induced increased kinase activity of c-Kit, suggesting an increased protein level of c-Kit [25]. As we were unable to detect an increase in the mRNA level of c-Kit following BMP4 treatment of our cultured mouse SSCs, BMP4 seems to regulate the translation instead of transcription of c-Kit. Our observation that the mRNA levels of c-Kit and Stra8 were upregulated by RA was consistent with the study by Zhou et al. [68]. More importantly, pretreatment of SSCs with BMP4 could increase the mRNA expression of Stra8 and c-Kit to significantly higher levels compared with the RA only group. BMP4 by itself is probably unable to induce spermatogonial differentiation but just prepares the cells to be responsive to other environmental signal(s) such as RA, the production of which is regulated in a rather complex manner* in vivo* [69, 70]. Otherwise, the SSCs pool could be exhausted shortly after birth because of the differentiation-inducing signals of the autocrine BMP4. The present study offers new insights into understanding the roles of autocrine and environmental factors that regulate the differentiation of SSCs during mammalian spermatogenesis.

## Supplementary Material

Supplemental Data Supplemental Data including 4 figures can be found with this article online.

## Figures and Tables

**Table 1:** PCR primer sequences and product size.

Gene	Forward primer (5′–3′)	Reverse primer (5′–3′)	Product size
BMP4	TCGTTACCTCAAGGGAGTGG	GGCGACGGCAGTTCTTATTC	159 bp
BMPR1A	GGGTCGTTACAACCGTGAT	CAACCTGCCGAACCATCT	164 bp
BMPR1B	TCAATGTCGTGACACTCCCATTCCT	TGCTGTACCGAGGTCGGGCT	245 bp
ActRIIB	CGAGCGCTTCACCCACTTG	CACCACGACACCACGGCAC	650 bp
ActRII	CCGGAGATGGAAGTCACA	CACATCCACACTGGTGCC	432 bp
BMPRII	AGGCCCAATTCTCTGGATCT	CACTGCCATTGTTGTTGACC	207 bp
Actin	CAGCCTTCCTTCTTGGGTAT	TGGCATAGAGGTCTTTACGG	100 bp
Stra8	ACAAGAGTGAGGCCCAGCAT	CCTCTGGATTTTCTGAGTTGCA	71 bp
c-Kit	GCCACGTCTCAGCCATCTG	GTCGGGATCAATGCACGTCA	119 bp
