# Supplementary material for: BMP4 Cooperates with Retinoic Acid to Induce the Expression of Differentiation Markers in Cultured Mouse Spermatogonia
Source: Stem Cells Int. 2016 Oct 4;2016:9536192. doi: 10.1155/2016/9536192 (PMC5067322; doi:10.1155/2016/9536192)

# Supplementary materials

**Figure S1. Establishment of spermatogonial cultures.**

(A) Total testicular cells from DBA/ICR-green F1 mice were cultured for 48 hours, spermatogonia (red arrow) loosely attached to the somatic cells (upper panel) were named as P0 culture, which were then collected by gentle pipetting and passaged onto MEF feeder and cultured for another 48 hours (P1) (middle panel). Spermatogonia formed colonies on MEF feeder (red arrow) and only few somatic cells left (red arrow head). After passaging to P2 (lower panel), spermatogonia began to form stable colonies on MEF feeder (red arrow) and almost all the somatic cells were removed. (B-C) Immunofluorescent analysis of P3 (B) and P33 (C) *in vitro* cultured spermatogonia using antibodies against SSCs markers DAZL and RET. Scale bars indicate 20 μm.

**Figure S2.** **High resolution fluorescent pictures showing the localization of transplanted green germ cells at different differentiating stages in recipient mouse seminiferous tubules**. Scale bar indicates 20 μm.

**Figure S3. Exogenous BMP4 does not affect the expression of Stra8 and c-Kit in cultured spermatogonia.** (A-B) Realtime PCR analysis of Stra8 expression in cultured spermatogonia stimulated with BMP4 at indicated concentration for 24 hours (A) and 48 hours (B). (C-D) Realtime PCR analysis of c-Kit expression in cultured spermatogonia stimulated with BMP4 at indicated concentration for 24 hours (C) and 48 hours (D). The data were presented as mean ± SD of three independent experiments.

**Figure S4. Purification of spermatocytes and round spermatids from adult mouse testis.** (A) Pre-purified adult mouse whole testis cells. (B) Purified adult mouse spermatocytes. (C) Purified adult mouse round spermatids. Scale bars indicate 20 μl.

Figure S1


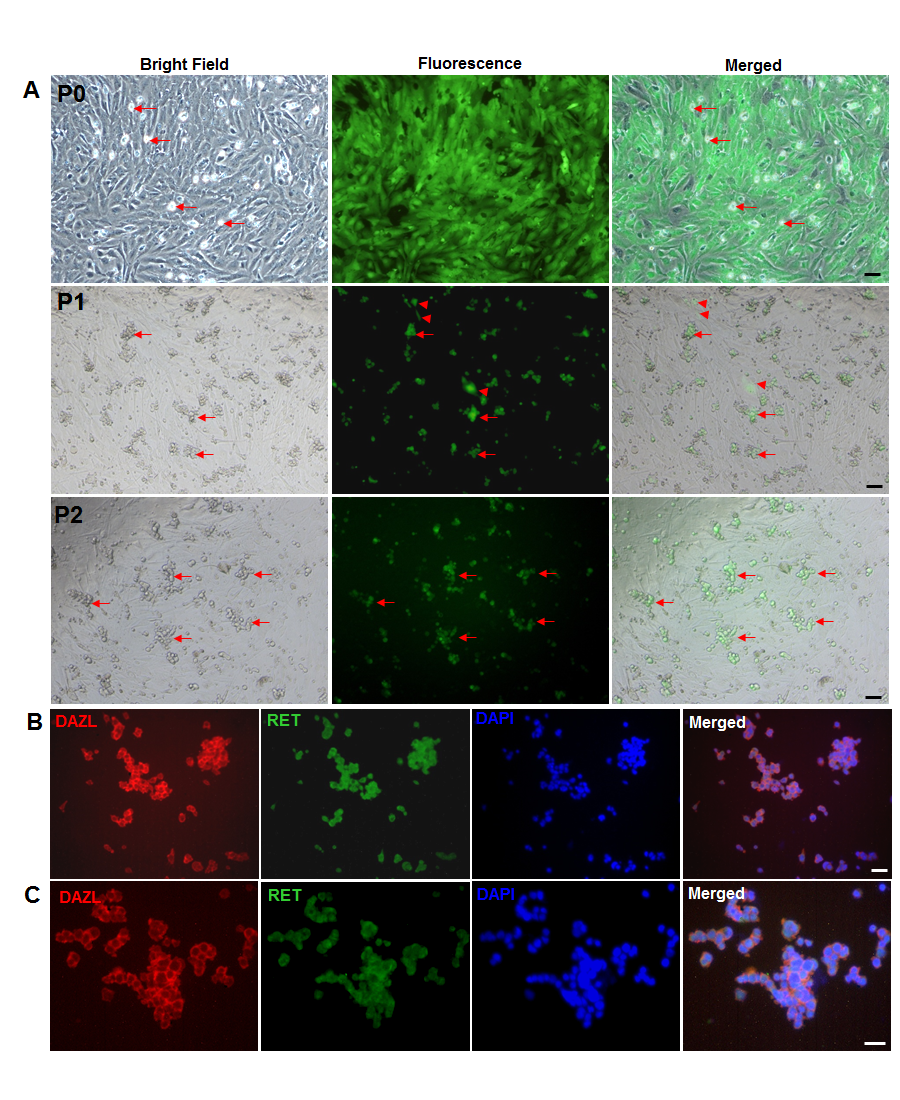


Figure S2


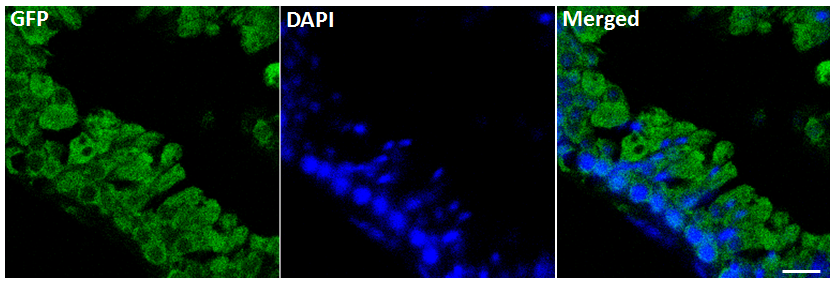


Figure S3


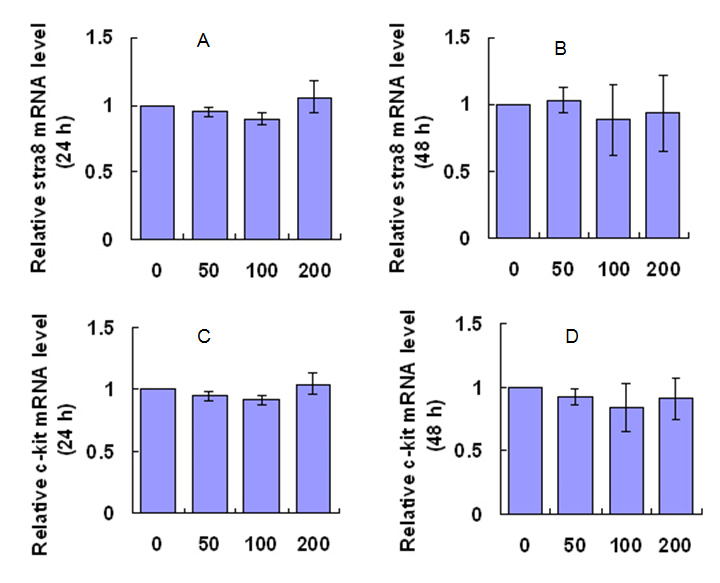


Figure S4


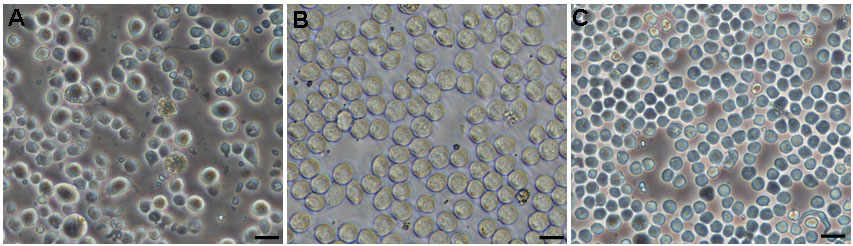

Supplement: Supplementary file 1 — Supplemental Data Supplemental Data including 4 figures can be found with this article online. [file 9536192.f1.docx]
